# High Salt Remodels Kidney Metabolism: Metabolite Fuel, Fate, and Signals

**DOI:** 10.1093/function/zqae006

**Published:** 2024-01-29

**Authors:** Moritz Lassé, Markus M Rinschen

**Affiliations:** III. Department of Medicine, University Medical Center Hamburg-Eppendorf (UKE), Hamburg 20246, Germany; Hamburg Center for Kidney Health (HCKH), University Medical Center Hamburg-Eppendorf, Hamburg 20246, Germany; III. Department of Medicine, University Medical Center Hamburg-Eppendorf (UKE), Hamburg 20246, Germany; Hamburg Center for Kidney Health (HCKH), University Medical Center Hamburg-Eppendorf, Hamburg 20246, Germany; Department of Biomedicine, Aarhus University, Aarhus 8000, Denmark; Aarhus Institute of Advanced Studies (AIAS), Aarhus 8000, Denmark

## A Perspective on “Metabolic Responses of Normal Rat Kidneys to a High Salt Intake”

The first line of therapy for many cardiovascular and renal diseases is lifestyle changes that involve low-salt diets.^1,2^ A low-salt diet benefits renal function, particularly in people with salt-sensitive hypertension. The adoption of a low-sodium diet could potentially improve albuminuria, hypertension, fluid status, and arterial stiffness, particularly for salt-sensitive individuals. The mechanism of salt handling and adaptation of the kidney, and their impact on kidney-controlled physiological circuits, are—in broad strokes—well understood. However, we still lack a detailed understanding of what a salt load means for kidney metabolism. Emerging studies now begin to illuminate how salt rewires kidney metabolism.

Kidneys are crucial metabolic organs, and they are very altruistic. Kidneys filter, reabsorb, secrete, and metabolize vast amounts of small molecules. This forms the basis of physiological homeostasis of the body.^3^ The kidney needs to balance the energy expenditure needed to fuel this metabolic function. Any disruption of normal metabolic functionality may—ultimately—lead to a loss of detoxification, accumulation of waste products in the system, and is, in part, responsible for the high overall mortality and morbidity associated with chronic kidney disease. Thus, enhancing our comprehension of both intrarenal metabolism and the role of renal metabolism in maintaining overall body homeostasis is essential.

Challenging the kidney with high salt (HS) leads to alterations in metabolism. An increased transport load would require more energy expenditure in the form of ATP usage to conserve sodium and fluids. Consistently, a linear increase in tubular sodium load leads to a linear increase in oxygen consumption.^4^ This ATP needs to be generated through burning of fuel. This fuel includes a variety of metabolites such as pyruvate, lactate, tricarboxylic acid intermediates such as citrate and alpha-ketoglutarate, amino acids, and fatty acids. All these metabolites form acetyl-coenzyme A, which, in turn, fuels the respiratory oxidation in the mitochondria.

In their study in *FUNCTION*, Shimada and colleagues^5^ present an elegant study using a sophisticated setup allowing the authors to gain novel insights under physiological conditions. The study used normal Sprague-Dawley (SD) rats that were fed a HS diet, specifically 4.0% NaCl, to investigate the effects of salt intake on kidney metabolism. The studies’ unique approach is that the authors carefully examined the metabolic contributions of the kidney, by investigating urine and plasma (both arterial and renal venous) metabolite context, and integrating this with kidney oxygen consumption and intrarenal metabolic and transcriptomic signatures. With their marriage of state-of-the-art physiology with relevant large-scale omics-based tools, the authors now provide a rich resource to mine and understand the physiological responses to HS diet. The major contribution of this study is the understanding of metabolic contributions of the kidney, as derived from their integration of arterial and renal venous metabolite levels. Thus, the authors provide the first window into the metabolic contributions of the kidney to overall body homeostasis in vivo.

Intriguing signals emerge regarding the metabolic contribution of the kidney—as measured by both arterial and venous metabolic signals. For instance, in this study, a net release of several amino acids into the bloodstream under high-salt challenge—as compared to baseline conditions—was observed in renal venous plasma. This included, for instance, phenylalanine, an essential amino acid for humans. This could either suggest that more phenylalanine is being reabsorbed by the proximal tubules, or that renal metabolization of phenylalanine is reduced. Aromatic amino acid metabolites that could be derived of phenylalanine, such as cinnamoyl compounds and cresol-related compounds, are linked to cardiovascular diseases.^6^ The authors make further interesting observations, such as a strong signal for lysine metabolism, although not all lysine degradation products are covered by the authors’ platform.^7^ These exciting observations add to the intriguing perspective that renal amino acid metabolism is a driver of salt response, and potentially even salt-sensitive hypertension.^7,8^

Challenges in the interpretation of the data are inherent to the nature of the omics data, and require further scrutinization—as also acknowledged by the authors. A prime example is the authors’ interpretation regarding the occurrence of the Warburg-like effect, hypothesized based on an increased abundance of pyruvate and lactate in the cortex. Transcriptomic pathway-level analyses seem to suggest such a Warburg-like effect, the occurrence of a glycolytic shift in the kidney. Here, the authors’ interpretation remains—to a certain degree—speculative. One could argue that RNA transcript data does not reflect protein abundance, and that protein phosphorylation is probably a more essential driver of the metabolic flux. It is even more challenging to attribute increased lactate to glycolysis. Lactate is an end product of glycolysis—an energy production pathway rarely done in the proximal tubule. Additionally, lactate is a substrate for gluconeogenesis, with enzymes being shared between glycolysis and gluconeogenesis. Lactate is also fuel for the tricarboxylic acid cycle in many tissues, including the kidney.^3^ Fate analyses—using stable isotopes—would allow the monitoring of metabolite conversions and determine contributions to pathway fluxes, and metabolic circuits. A metabolic switch to anaerobic glycolysis is not confirmed by the experiments, but also not ruled out and yet needs to be corroborated by independent and orthogonal studies. In fact, increased glycolysis (for which no oxygen is required) would not contribute to the observed salt-dependent increase in oxygen consumption.

Further, important signaling effects of metabolites that act beyond fueling the cells need to be considered in the author’s model. Some metabolite-driven signaling effects are directly linked to sodium handling of the kidney, for instance, succinate.^9^ Sex differences—with huge effects on kidney proximal tubule function—are also not considered or studied.^10^ These possibilities require closer analyses on the (phospho-)proteome and metabolic flux level.

In conclusion, Shimada and colleagues demonstrate elegantly the physiological impact salts can have on kidney and tubule metabolism and with that they open the door for many future investigators. High salt modulates the metabolic kidney function and further studies will lead to a better understanding of the detailed metabolic ramifications, including flux and signaling during HS challenge in the context of renal and cardiovascular function.

## References

[1] He FJ, Tan M, Ma Y, MacGregor GA. Salt reduction to prevent hypertension and cardiovascular disease: JACC state-of-the-art review. J Am Coll Cardiol. 2020;75(6):632–647. 32057379 10.1016/j.jacc.2019.11.055

[2] Kalantar-Zadeh K, Jafar TH, Nitsch D, Neuen BL, Perkovic V. Chronic kidney disease. Lancet. 2021;398(10302):786–802. 34175022 10.1016/S0140-6736(21)00519-5

[3] Chrysopoulou M, Rinschen MM. Metabolic rewiring and communication: an integrative view of kidney proximal tubule function. Annu Rev Physiol. 2023;86(1). https://pubmed.ncbi.nlm.nih.gov/38012048/. 10.1146/annurev-physiol-042222-024724 38012048

[4] Lassen NA, Munck O, Thaysen JH. Oxygen consumption and sodium reabsorption in the kidney. Acta Physiol Scand. 1961;51(4):371–384. 13759307 10.1111/j.1748-1716.1961.tb02147.x

[5] Shimada S, Hoffmann BR, Yang C et al. Metabolic responses of normal rat kidneys to a high salt intake. Function (Oxf). 2023;4(5):zqad031. 37575482 10.1093/function/zqad031 PMC10413938

[6] Billing AM, Kim YC, Gullaksen S et al. Metabolic communication by SGLT2 inhibition. Circulation. 2023. https://pubmed.ncbi.nlm.nih.gov/38152989/. 10.1161/CIRCULATIONAHA.123.065517 PMC10922673 38152989

[7] Tan Y, Chrysopoulou M, Rinschen MM. Integrative physiology of lysine metabolites. Physiol Genomics. 2023;55(12):579–586. 37781739 10.1152/physiolgenomics.00061.2023

[8] Hou E, Sun N, Zhang F et al. Malate and aspartate increase L-arginine and nitric oxide and attenuate hypertension. Cell Rep. 2017;19(8):1631–1639. 28538181 10.1016/j.celrep.2017.04.071

[9] Peti-Peterdi J, Gevorgyan H, Lam L, Riquier-Brison A. Metabolic control of Renin secretion. Pflugers Arch. 2013;465(1):53–58. 22729752 10.1007/s00424-012-1130-y PMC4574624

[10] McDonough AA, Layton AT. Sex differences in renal electrolyte transport. Curr Opin Nephrol Hypertens. 2023;32(5):467–475. 37382185 10.1097/MNH.0000000000000909 PMC10526720

